# Involvement of Transcription Factor FoxO1 in the Pathogenesis of Polycystic Ovary Syndrome

**DOI:** 10.3389/fphys.2021.649295

**Published:** 2021-03-05

**Authors:** Renfeng Xu, Zhengchao Wang

**Affiliations:** Provincial Key Laboratory for Developmental Biology and Neurosciences, Provincial University Key Laboratory of Sport and Health Science, Key Laboratory of Optoelectronic Science and Technology for Medicine of Ministry of Education, College of Life Sciences, Fujian Normal University, Fuzhou, China

**Keywords:** forkhead transcription factor FoxO1, polycystic ovary syndrome, low-grade inflammatory response, insulin resistance, tumor necrosis factor alpha

## Abstract

FoxO1 is a member of the forkhead transcription factor family subgroup O (FoxO), which is expressed in many cell types, and participates in various pathophysiological processes, including cell proliferation, apoptosis, autophagy, metabolism, inflammatory response, cytokine expression, immune differentiation, and oxidative stress resistance. Polycystic ovary syndrome (PCOS) is the most common endocrine disorder in the women of childbearing age, which is regulated *via* a variety of signaling pathways. Currently, the specific mechanism underlying the pathogenesis of PCOS is still unclear. As an important transcription factor, FoxO1 activity might be involved in the pathophysiology of PCOS. PCOS has been associated with insulin resistance and low-grade inflammatory response. Therefore, the studies regarding the role of FoxO1 in the incidence and associated complications of PCOS will help provide novel ideas for establishing the treatment strategy of PCOS.

## Introduction

At present, the incidence rate of polycystic ovary syndrome (PCOS) is about 5.6% among women of reproductive age (19–45 years) in Chinese Han population based on a large community-based study (Li et al., 2013), but the specific mechanism underlying the pathogenesis of PCOS is still unclear. Apart from polycystic ovaries, hyperandrogenism, and ovulatory disorders, PCOS is often accompanied by insulin resistance (IR), low-grade chronic inflammatory response, obesity, abnormal lipid metabolism, and long-term complications, such as hypertension, type 2 diabetes, and endometrial cancer (Li et al., 2013; Barthelmess and Naz, 2014; Nandi et al., 2014; Wang et al., 2015, 2017a,b; Wang and Wang, 2017; Lin et al., 2019; Zhang et al., 2019).

Forkhead transcription factor subfamily O (FoxO) widely exists in various mammalian tissues and plays an important role in metabolism, cell proliferation, apoptosis, and stress resistance, while FoxO1, a member of FoxO, has been shown to play a vital role during glycolipid metabolism, IR, and oxidative stress (Wang et al., 2016; Lee and Dong, 2017; Murtaza et al., 2017). Previous studies indicate that hepatic IR involves ceramide-induced activation of atypical protein kinase C, which selectively impairs protein kinase B (PKB/Akt)-dependent FoxO1 phosphorylation (Sajan et al., 2014, 2015). In granulosa cells (GCs) derived from PCOS patients and the ovarian tissues of PCOS rats, the expression levels of insulin growth factor 1 (IGF-1R) and Wnt family member 1 (Wnt1) were found to be decreased and PKB/Akt^Ser473/Thr308^ phosphorylation was lowered (Mao et al., 2018). Recent research demonstrated that Cangfudaotan decoction alleviated IR and improved follicular development in rats with PCOS via IGF-1-PI3K/Akt-Bax/Bcl-2 pathway (Wang et al., 2020). Advanced glycation end product-induced apoptosis involves the formation of reactive oxygen species, nitric oxide, and ceramide, and further leads to p38 and JNK mitogen-activated protein kinase (MAPK) activation, which in turn induces FoxO1 and caspase-3 (Alikhani et al., 2007). MicroRNA-145 (miR-145) mimics inhibit the activation of p38 MAPK and extracellular signal-regulated kinase through targeting insulin receptor substrate 1 (IRS1), and overexpressed IRS1 abrogated this suppressive effect in the GCs derived from PCOS patients (Cai et al., 2017). *Klotho* gene knockdown blocked the effects of insulin on apoptosis/proliferation in the GCs derived from PCOS patients, and inhibited caspase-3 activity in the ovarian tissues of PCOS rats (Mao et al., 2018). Interleukin-1β (IL-1β)-dependent regulation of FoxO1 protein content and its localization in a novel ceramide-dependent manner through IL-1β stimulation of primary rat hepatocytes and in HEK293 cells overexpressing IL-1β receptor have been demonstrated previously (Dobierzewska et al., 2012). Yang et al. (2020) found that cryptotanshinone (CRY) significantly alleviated the changes in the body and ovary weight, and the levels of hormone and inflammatory factor in PCOS rats through regulating the HMGB1/TLR4/nuclear factor-kappa B (NF-*κ*B) signaling pathway. Furthermore, the upregulation of miR-204 improved IR of PCOS *via* the inhibition of HMGB1 and inactivation of the TLR4/NF-κB pathway (Jiang et al., 2020), while increased HMGB1 and reduced FOXO1 were found to be dependent on the loss of cystic fibrosis (CF) transmembrane conductance regulator (CFTR) function in case of CF (Smerieri et al., 2014; Montanini et al., 2016b; Cirillo et al., 2019b).

**Figure 1:**
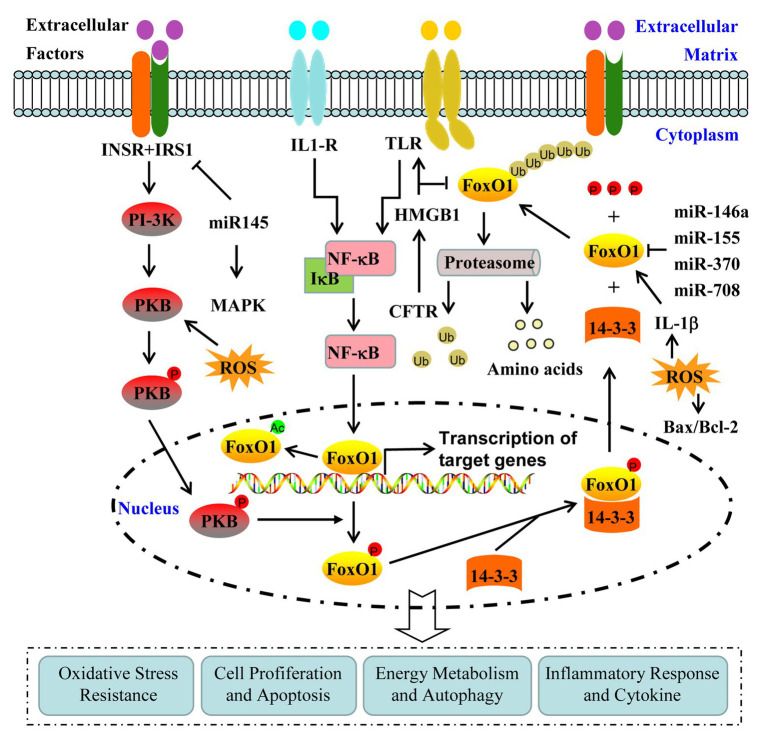
Regulation and contribution of FoxO1 activity in the pathogenesis of polycystic ovary syndrome (PCOS). FoxO1 activity is mainly regulated by the post-translational modifications, including phosphorylation, acetylation, and ubiquitination. FoxO1 is involved in the pathogenesis of PCOS through various signaling pathways, including phosphoinositide 3-kinase (PI-3K)/protein kinase B (PKB), mitogen-activated protein kinase (MAPK), high-mobility group box 1(HMGB1)/Toll-like receptor 4(TLR4)/nuclear factor-kappa B (NF-κB), and Interleukin-1β (IL-1β).

At present, the investigation on the contribution of FoxO1 in the pathogenesis of PCOS is being conducted. Therefore, exploring the underlying mechanism of FoxO1 activity in the pathogenesis of PCOS will help provide a novel target for establishing the treatment of PCOS and associated complications (Figure 1).

## PCOS Pathogenesis

The origin of PCOS is multifactorial with individual differences, such as abnormal ovarian steroid secretion, hyperinsulinemia, increased luteinizing hormone, and other aspects, which lead to complementary or synergistic effects, and affect the development of the disease (Wang and Wang, 2017) and, therefore, the exact cause of PCOS is still unclear. Given the polymorphism of PCOS phenotype, it is considered to be a multi-gene-mediated disease (Li et al., 2013; Barthelmess and Naz, 2014; Nandi et al., 2014; Wang and Wang, 2017). For example, PCOS has previously been related to insulin receptor (INSR) gene with racial differences (Stewart et al., 2006; Wang et al., 2017a; Lin et al., 2019), and the family history might be a potential risk factor for the incidence of PCOS (Azziz and Kashar-Miller, 2000; Wang and Wang, 2017). It has been also reported that visceral obesity, proinflammatory factors, hyperinsulinemia, and IR are likely associated with the occurrence of PCOS (Wang et al., 2015, 2017b; Hughan et al., 2016; Zhang et al., 2019).

Polycystic ovary syndrome patients with hyperinsulinemia or IR are not dependent on obesity, body fat distribution, and androgen levels, and the risk of impaired glucose tolerance and type 2 diabetes mellitus is higher in these patients than the normal individuals (Nandi et al., 2014). The high androgen level and occurrence of IR in PCOS patients might be related to the continuous release of inflammatory factors from adipose tissue (Barthelmess and Naz, 2014; Wang and Wang, 2017; Cirillo et al., 2019a; Barber and Franks, 2021). A large number of studies have demonstrated a role of inflammation in the pathogenesis of PCOS (Alikhani et al., 2007), and the association of increased inflammatory markers, such as C reactive protein (CRP), ferritin, tumor necrosis factor (TNF) alpha, interleukin-6 (IL-6), and interleukin-18 (IL-18) with the occurrence of PCOS (González et al., 2006, 2007, 2012). Increased levels of plasminogen activator inhibitor-1 (PAI-1) and free fatty acid affect the phosphorylation of serine residue, leading to IR. PCOS patients exhibit high levels of ferritin and transferrin of hemoglobin along with a decrease in the levels of anti-inflammatory cytokines and anti-oxidant factors, thereby leading to chronic inflammation (Escobar-Morreale and Luque-Ramírez, 2011; Escobar-Morreale, 2012; Yang et al., 2015). Therefore, obesity may increase the level of oxidative stress in adipose tissue, activate the inflammatory signaling, and finally aggravate the chronic inflammatory state and IR in PCOS patients (Furukawa et al., 2004).

## Structure and Function of Foxo1

The forkhead protein family was discovered in a study regarding the abnormal head mutations of Drosophila in 1989, which contained a highly conserved DNA binding domain, which corresponds to the forkhead conserved region composed of 110 amino acid residues and the domains of three helixes, three folds, and two wing-like structures (Weigel et al., 1989). At present, more than 100 forkhead (FOX) genes have been identified, belonging to 19 subfamilies, namely FOXA~S (Genin et al., 2014). The subgroup O of FoxO is the earliest discovered and widely distributed subgroup, which comprises FoxO1, FoxO3, FoxO4, and FoxO6 (van der Vos and Coffer, 2011). The first two are expressed in almost all human tissues, while FoxO4 is mainly expressed in muscles, kidney, and colon tissues, and FoxO6 is expressed in the brain and liver (van der Vos and Coffer, 2011).

The function of transcription factor FoxO1 is complex, which is mainly through the activation or inhibition of the transcription of its downstream target genes (Xu et al., 2017; Xing et al., 2018). FoxO1 in the endometrium has been shown to play an important role in the transformation of endometrium during menstruation, and in the protection of fetal mothers from oxidative damage during pregnancy (Kajihara et al., 2013). Moreover, FoxO1 knockout leads to embryo death due to vascular dysplasia (Hosaka et al., 2004). It has been reported that mice with specific loss of FoxO1 in liver can resist IR induced by high-fat diet, while those with specific over-expression of FoxO1 in liver can increase IR (Kim et al., 2009; Balakumar et al., 2016; Pandey et al., 2017; Zeng et al., 2019). The acute inflammation process is related to the increase in glucocorticoid production activated *via* the FoxO1 pathway, and then, glucocorticoid reduces insulin-like growth factor 1 (IGF-1) production and increases TNF alpha/NF-kB signaling during the induction of protein hydrolysis system (Kim et al., 2009; Schakman et al., 2012).

## Regulation of Foxo1 Activity

The transcriptional activity of FoxO1 is mainly accomplished through complex post-translational modifications, including phosphorylation, acetylation, and ubiquitination. These modifications can be activating or inactivating. The activity of specific targets can be altered through four functional sequences, thereby resulting in different biological effects (Tsai et al., 2007).

Phosphorylation of FoxO1 is directly by several protein kinases, which can modify different sites of this transcription factors through changing their subcellular location, DNA binding affinity, and transcriptional activity (Zhao et al., 2004; Tikhanovich et al., 2013). FoxO1 is phosphorylated through the activation of the serine-threonine kinases, including PKB/AKT and serum glucocorticoid inducible kinase (SGK), by phosphatidylinositol-3 kinase (PI-3K) to associate FoxO1 with 14-3-3 couple protein binding for translocating from the nucleus to the cytoplasm, thereby resulting in its transcription inactivation (Wang et al., 2016). Furthermore, growth factor-activated protein kinases, such as extracellular signal-regulated kinase and cyclin-dependent kinase-2, also induce FoxO1 phosphorylation and its transport to the cytoplasm through different pathways, thereby resulting in a decrease in FoxO1 transcriptional activity (Zhao et al., 2004).

Acetylation of FoxO1 promotes and decreases the transcriptional activity of FoxO1, which is mediated through histone acetyltransferase and deacetylase, thereby regulating different biological functions (Lalmansingh et al., 2012). FoxO1 regulates the affinity and sensitivity of DNA binding regions through the acetylation of K262, K265, K274, and K294, thereby altering downstream PKB/AKT phosphorylation (Calnan and Brunet, 2008; Lalmansingh et al., 2012). Additionally, FoxO1 also reduces its own activity through acetylating two basic residues, Lys242, and Lys245, in the carboxyl terminal of DNA-binding region of cAMP responsive element binding protein (Daitoku et al., 2004).

Unlike the reversibility of phosphorylation/dephosphorylation and acetylation/deacetylation of FoxO, ubiquitination of FoxO1 is irreversible, and thus, is responsible for the degradation of FoxO1 (Huang and Tindall, 2011). Ubiquitin-protein ligating enzyme (E3) is a key enzyme for recognizing ubiquitin and degrading protein substrates. The degradation of FoxO1 is achieved *via* the multi-ubiquitination of multiple E3 complexes (Huang and Tindall, 2011).

## Foxo1 and PCOS

Polycystic ovary syndrome is a disease with an endocrine disorder and the development of PCOS may be caused due to the imbalance in the levels of sex hormones, inflammatory factors, and insulin. Notably, FoxO1 expression was found to be increased significantly in cumulus cells of PCOS women with BMI 21.5 ± 2.5 kg/m^2^ than that in non-PCOS patients with BMI 20.7 ± 2.1 kg/m^2^ (Shi et al., 2015).

### FoxO1 and Insulin Resistance

Polycystic ovary syndrome patients with hyperinsulinemia or IR were reported to be about 44–77% (Vigil et al., 2007). IR is a state of pathological metabolism with decreased ability to use glucose, and thus, insulin secretion is increased to compensate and maintain the normal blood glucose level, thereby leading to hyperinsulinemia. Interestingly, hyperinsulinemia not only increases androgen secretion through selectively increasing the sensitivity of theca cells to luteinizing hormone, but also increases the level of free androgen through inhibiting the synthesis of sex hormone binding globulin in the liver, thereby promoting the occurrence of PCOS (Bremer and Miller, 2008; Wang et al., 2015, 2017a,b; Lin et al., 2019; Zhang et al., 2019).

FoxO1 is a key downstream molecule of the INS/IGF-1 signaling pathway, regulating the circulatory metabolism and hormone levels in liver, pancreas, hypothalamus-pituitary axis, and adipose tissue through increasing the level of circulating glucose (Wang et al., 2015, 2017a,b; Kamagate and Dong, 2018; Lin et al., 2019; Zhang et al., 2019). For example, FoxO1 elevates the blood glucose levels through acting on the key enzymes, such as glucose-6-phosphatase and phosphoenolpyruvate carboxykinase during the process of gluconeogenesis, and it also affects the apoptosis of beta cells and development of type 2 diabetes mellitus through INS/IGF-1 signaling (Wang et al., 2015, 2017a,b; Kamagate and Dong, 2018; Lin et al., 2019; Zhang et al., 2019). Rosas et al. (2010) found that the expression of glucose transporter 4 (GLUT4) related molecules in endometrium during secretory phase of normal menstrual cycle was beneficial for glucose uptake, while some molecules in PCOS patients related with hyperandrogenism decreased, and the exposure of GLUT4 and absorption of glucose reduced, thereby resulting in IR. Kohan et al. (2010) found that the decrease in GLUT4 expression in endometrium of PCOS patients with IR was related to FoxO1 phosphorylation, indicating that FoxO1 phosphorylation inhibited the expression of GLUT4 gene, and thus, affected the function of endometrium and caused IR.

### FoxO1 and Chronic Inflammation

The expression of several chronic inflammatory factors was found to be increased in PCOS patients, including CRP, IL, and TNF alpha. These inflammatory factors reduce the sensitivity of tissue cells to insulin through endocrine, paracrine, and autocrine mechanisms, thereby leading to IR (González et al., 2006, 2007, 2012; Escobar-Morreale et al., 2011). Conversely, there is a common pathway between the signal transduction of inflammatory factors and INSR. Inflammatory factors can directly interfere with the phosphorylation of INSR, thereby changing the downstream pathway, leading to IR. Conversely, some inflammatory factors can increase the expression of rate-limiting enzymes that catalyze steroid production in theca cells and increase androgen levels in PCOS patients.

González et al. demonstrated that the expression of NF-kB increased in PCOS patients with high blood glucose, and the increased activity of NF-kB resulted in the secretion of pro-inflammatory cytokine TNF alpha (González et al., 2006, 2012; Escobar-Morreale et al., 2011). TNF alpha induces lipolysis of visceral fat, releases free fatty acids, and eventually leads to IR and hyperandrogenism (González et al., 2006, 2007, 2012; Escobar-Morreale et al., 2011). Elevated androgen may change the local expression of androgen receptor in the ovaries, and then increase the occurrence of PCOS (González et al., 2006, 2012; Escobar-Morreale et al., 2011). Ibfelt et al. (2014) found that TNF alpha induces IR through inhibiting the tyrosine phosphorylation of insulin receptor substrates, and also affects the intracellular glucose transport through downregulating the expression of GLUT4. Miao et al. (2012) found that TNF alpha is positively correlated with FoxO1 expression and FoxO1 might increase the production of pro-inflammatory factors in diabetic hepatocytes with IR. Li et al. (2017) found the association of FoxO1 signaling with the aggravation of inflammation and occurrence of IR in PCOS macrophages.

### FoxO1 and Obesity

During the last four decades, obesity has driven the rise in obesity-related co-morbidities, including PCOS (Barber and Franks, 2021). PCOS is associated with IR, which is independent of (but amplified by) obesity (Barber and Franks, 2021). Multiple factors contribute to the severity of IR in PCOS, including most notably, weight gain (Barber and Franks, 2021). In the study conducted by Šimková et al. (2020), the authors demonstrated that there were no differences in hormonal, but in metabolic parameters, between normal-weight and obese PCOS women. Obese PCOS women exhibited significantly higher IR, fatty-liver index, triglycerides, and cytokines (IL-2, IL-13, and IFN-gamma; Šimková et al., 2020). Ni et al. (2015) found that the expression level of high-mobility group box 1 (HMGB1) was increased in the serum from PCOS women with IR/hyperinsulinemia. Further investigation discovered that the high concentration of insulin not only mimicked IR model, but also promoted apoptosis of ovarian GCs through HMGB1 (Ni et al., 2015). Montanini et al. (2016a) also found that HMGB1 expression was increased in CF patients with deranging glucose metabolism. The increase in HMGB1 was related to the loss of CFTR function, and insulin lowered HMGB1 (Montanini et al., 2016a). CFTR inhibitor and siRNA experiments demonstrated that the changes in FoxO1 were also related to CFTR loss of function in CF (Smerieri et al., 2014), and reduced FoxO1 is correlated with reduced gluconeogenesis and increased adipogenesis, which are the characteristic features of insulin insensitivity (Smerieri et al., 2014). In PCOS women with BMI 25.92 ± 0.99 kg/m^2^, CFTR and FoxO1 expression levels reduced in GCs (Cirillo et al., 2019b), and HMGB1 expression increased in follicular fluids and serum of PCOS women (Cirillo et al., 2019b). Additionally, miRNAs analyzed by Cirillo et al. (2019a) demonstrated the changes in PCOS ovaries and their relationships with inflammation and insulin sensitivity. Montanini et al. also found that significant changes in the expression of these four miRNAs (miR-146a, miR-155, miR-370, and miR-708) were dependent on the genotype and glucose tolerance state in CF patients (Cirillo et al., 2019a), which were selected as the potential FoxO1 regulators (Cirillo et al., 2019a). Cai et al. (2017) found that IRS1 gene is a direct target of miR-145, which was downregulated in GCs derived from PCOS patients. Further analysis demonstrated that miR-145 mimics inhibited cell proliferation and promoted apoptosis in GCs derived from PCOS women (Cai et al., 2017).

## Summary and Conclusion

In conclusion, FoxO1, as a crucial transcription factor, plays an important role in regulating the gene expression, participating in gluconeogenesis, low-density lipoprotein production, oxidative stress, and cell apoptosis (Weigel et al., 1989; Furukawa et al., 2004; Escobar-Morreale and Luque-Ramírez, 2011; Escobar-Morreale, 2012; Kajihara et al., 2013; Genin et al., 2014; van der Vos and Coffer, 2011; Yang et al., 2015; Wang et al., 2016; Lee and Dong, 2017; Murtaza et al., 2017; Xu et al., 2017; Xing et al., 2018). Additionally, many studies have demonstrated that FoxO1 plays an important role in the pathogenesis of PCOS. The changes in the levels of hormones, TNF alpha, and GLUT4 in PCOS patients may affect the regulation of FoxO1 signaling on glucose transport, thereby leading to IR (Huang and Tindall, 2011). Moreover, the changes in FoxO1-mediated signaling may further induce the occurrence of low-grade chronic inflammation in the body, thereby leading to the hyperandrogenism of PCOS (Bremer and Miller, 2008; Rosas et al., 2010; Barthelmess and Naz, 2014; Nandi et al., 2014; Kamagate and Dong, 2018). Therefore, the study regarding the association of FoxO1 with the pathogenesis of PCOS can provide a basis for the etiology of PCOS, and a novel theoretical support for establishing the treatment of PCOS.

## Author Contributions

The manuscript was written by RX and revised by ZW. Both authors reviewed and approved the final version of the manuscript.

### Conflict of Interest

The authors declare that the research was conducted in the absence of any commercial or financial relationships that could be construed as a potential conflict of interest.
